# Long-term outcome after combined kyphoplasty and intraoperative radiotherapy (Kypho-IORT) for vertebral tumors

**DOI:** 10.1186/s13014-020-01715-z

**Published:** 2020-11-12

**Authors:** Frederic Bludau, Laura Winter, Grit Welzel, Udo Obertacke, Frank Schneider, Frederik Wenz, Arne Mathias Ruder, Frank A. Giordano

**Affiliations:** 1 Department of Orthopedic Surgery and Trauma Surgery, University Medical Center Mannheim, Mannheim Medical Faculty, Heidelberg University, Mannheim, Germany; 2 Department of Radiation Oncology, University Medical Center Mannheim, Mannheim Medical Faculty, Heidelberg University, Mannheim, Germany; 3 University Medical Center Freiburg, Freiburg University, Freiburg, Germany; 4 Department of Radiation Oncology, University Hospital Bonn, University of Bonn, Bonn, Germany

**Keywords:** Kypho-IORT, Electronic brachytherapy, Intraoperative radiotherapy, Kyphoplasty, Spine, Vertebral metastases

## Abstract

**Introduction:**

The spine represents the site which is most frequently affected by bone metastases in patients with systemic cancer. Of all local treatment options, combined kyphoplasty and intraoperative radiotherapy (Kypho-IORT) provides both, instantaneous stabilization and immediate pain relief. We here report on the long-term outcomes of the largest cohort treated with Kypho-IORT to date.

**Methods:**

Between 2009 and 2019 a total of 104 patients underwent Kypho-IORT to vertebral tumors in the thoracic, lumbar, or sacral spine with transpedicular kyphoplasty and intraoperative irradiation with a needle-shaped electronic brachytherapy source at our center. Patients were treated either *on trial*, within the prospective Kypho-IORT studies (NCT01280032 and NCT02773966), or, after completion of the study, *off trial* but compliant with the study protocol. Follow-up and imaging with computed tomography (CT) or magnetic resonance imaging was scheduled after 3 and 6 months and then bi-annually.

**Results:**

A total of 143 vertebrae (89 thoracic spine, 53 lumbar spine, and 1 sacral spine) were treated in 104 patients. The median follow-up was 14.5 months (range 0.4–109). Local progression occurred in 10 patients (10 vertebrae) after a median time of 22.3 months (range 1.5–73) resulting in local control rates of 97.1, 95.9, and 94.2% at 6, 12, and 24 months, respectively. Overall survival was 74.6, 61.7, and 50.3% at 6, 12, and 24 months, respectively. A single serious adverse event was reported.

**Conclusion:**

In addition to immediate pain reduction and stabilization, Kypho-IORT shows excellent long-term local control with minimal side effects.

## Introduction

The spine is the most common site for the occurrence of bone metastases [1]. Additionally, intraosseous hemangiomas frequently occur as benign tumors of the spine [2]. Treatment should focus on palliation of pain, stabilization and local tumor control with therapeutic options including radiation therapy, surgical intervention or radiofrequency ablation [3–5]. Due to continuous advancements in systemic cancer therapy, any delay or pause of systemic treatment caused by local interventions should be avoided. With the advent of advanced therapies, life expectancy of cancer patients continues to rise and local control as well as quality of life gain further importance [6–8].

Kypho-IORT consists of cement augmentation kyphoplasty and intraoperative irradiation as a “one-stop-shop” intervention for the treatment of tumors in vertebral bodies of the thoracic, lumbar, and sacral spine. In a recent dose escalation and cohort expansion phase I/II trial with a total of 61 patients we showed a significant median pain reduction at the first postoperative day and a subsequent sustained pain reduction. The 3-, 6-, and 12-month local progression free survival (L-PFS without considering death as an endpoint) was 97.5, 93.8, and 93.8% [9]. To further investigate this combined approach, a multi-centric randomized phase III trial (NCT02773966) was designed to test Kypho-IORT against external beam radiotherapy (EBRT) as standard-of-care for painful vertebral metastases [10]. We here report our long-term experience of 10 years with patients treated at our institution as the largest single-center cohort analysis to date.

## Methods

All prospective and retrospective data acquisition and analysis was approved by the local institutional review board. All patients treated with Kypho-IORT between 2009 and 2019 at our institution were included in this analysis, whereas treatment was either carried out *on trial* within the prospective Kypho-IORT studies (NCT01280032 and NCT02773966) or, after completion and closure of the phase I/II study or before the start of the phase III study, *off trial* but fully compliant with the study protocol. Patients with up to three painful and/or unstable metastases of pathologically confirmed cancer or benign tumors (hemangioma) in vertebral bodies (Tomita 1/2) of the thoracic, lumbar, and/or sacral spine were eligible for treatment. In brief, following a bi-pedicular approach and placement of guidance sleeves, a 50-kV X-ray source (Intrabeam, Carl Zeiss Meditec AG, Oberkochen, Germany) equipped with a needle applicator (diameter 4.2 mm) was inserted into the vertebral metastasis. A dose of 8 Gy was then prescribed to a distance of 8, 11, or 13 mm from the isocenter in the tip of the source. Following irradiation, transpedicular kyphoplasty with cement augmentation was performed [9]. Thereafter, patients continued with standard-of-care systemic therapy, depending on their primary cancer.

Baseline imaging was performed (computed tomography, CT, or magnetic resonance imaging, MRI) within 7 days after surgery. Follow-up spinal imaging was scheduled every 6 months, or in case new symptoms occurred (e. g. increased pain or neurological deficits). Median follow-up was defined by the time between Kypho-IORT and the last follow-up. Local recurrence was defined as tumor recurrence within a treated vertebra. Survival was measured as time frame from the intervention until death by any cause.

Local control rate (LC per treated vertebra) and overall survival rate (OS per patient) at 6, 12, 24, 36, 48, and 60 months were estimated using the Kaplan–Meier method. All statistical analyses were performed using SPSS (V. 24.0; IBM, Armonk, NY).

## Results

**Fig. 1:**
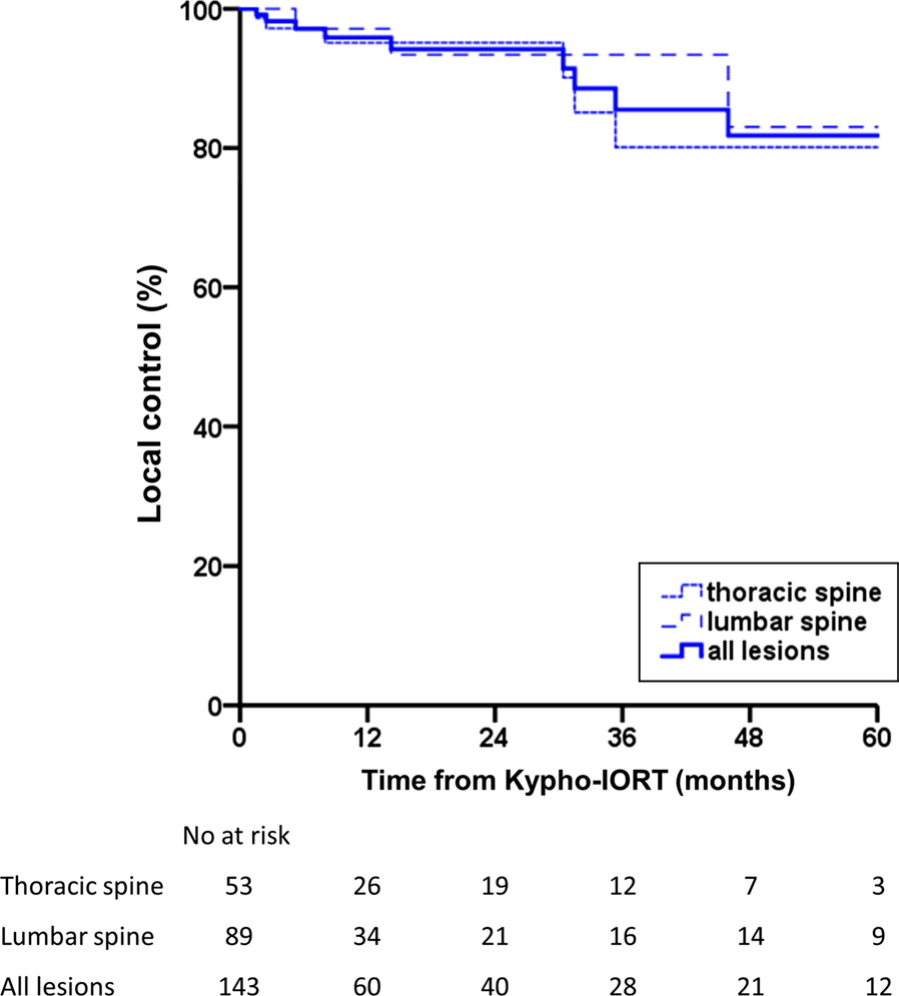
Local control after Kypho-IORT. Shown are the Kaplan–Meier plots for 89 vertebrae of the thoracic spine and 53 vertebrae of the lumbar spine as well as a combined plot of thoracic, lumbar, and sacral vertebrae treated with Kypho-IORT at our institution (the single patient with a treatment of 1 vertebra of the sacral spine died after 6 weeks and cannot be displayed as curve in the graph)

**Fig. 2:**
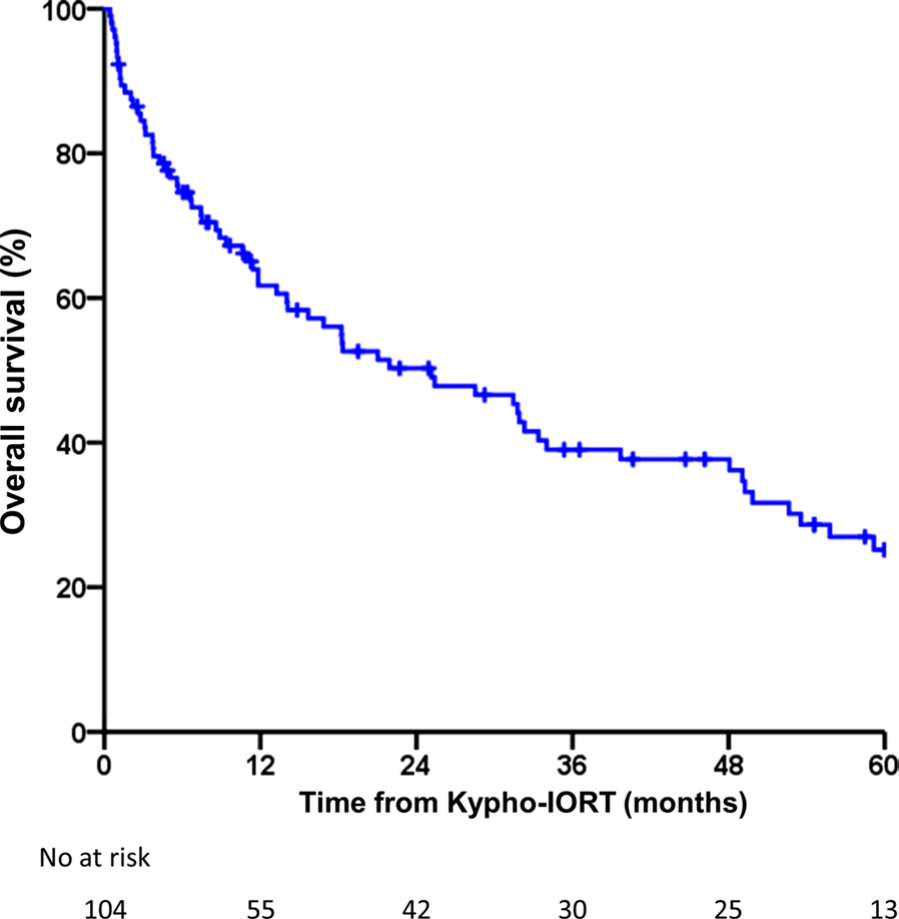
Overall survival after Kypho-IORT. Displayed is the overall survival of 104 patients as Kaplan–Meier plot

A total of 104 patients were included in this analysis. The median age at the time of Kypho-IORT was 62 years (range 32–85 years). 56% of the patients were female and 48% were male. A total of 143 lesions were treated, with 89 lesions located in the thoracic spine, 53 in the lumbar spine, and 1 in the sacral spine. Of these, 23 treatments were performed as 2-level interventions, and 8 as 3-level interventions. All vertebral bodies were intraoperatively irradiated with 8 Gy, which were prescribed to a distance from the isocenter of 8, 11, or 13 mm in 48, 20, and 75 vertebrae, accounting for 33.6, 14.0, and 52.4% of the vertebrae treated, respectively. The median follow-up was 14.5 months (range 0.4–109). We detected 10 local recurrences with 7 located in the thoracic spine and 3 located in the lumbar spine. Dose prescription distance to the recurrent lesions was 8, 11, and 13 mm in 2, 3, and 5 lesions, accounting for 4.2, 15, and 6.7% of recurrences in the 8, 11, and 13 mm subgroup, respectively. In one patient, a locally recurrent tumor in a pre-fractured vertebra caused a vertebral compression fracture (VCF). Median time to recurrence was 22.3 months (range 1.5–73). The underlying diseases of patients with local recurrence were breast (4), prostate (2), colorectal (1), renal (1) and lung cancer (1) or soft tissue sarcoma (1). The LC at 6, 12, 24, 36, 48, and 60 months for the thoracic spine was 97.2, 95.1, 95.1, 80.1, 80.1, and 80.1%, and for the lumbar spine 97.1, 97.1, 93.4, 93.4, 83.0, and 83.0%, respectively, with no recurrence in the sacral spine resulting in a combined LC of 97.1, 95.9, 94.2, 85.5, 81.8, and 81.8% (Fig. 1). No difference was detected for LC between the thoracic and the lumbar spine (log-rank test, *p* = 0.663). A total of 66 patients died, resulting in an OS at 6, 12, 24, 36, 48, and 60 months of 74.6, 61.7, 50.3, 39.0, 37.7, and 25.2% (Fig. 2). None of the mortalities were related to the procedure. A single serious adverse event (SAE) of temporary painful nerve root irritation immediately after surgery was noted. No adjacent level vertebral fractures occurred. Detailed data is reported in Table 1.

**Table 1:** Details of the cohort. Listed are the entities with the number of patients as well as vertebrae treated and the local recurrences per entity

Entities	Patients/proportion	Vertebrae/proportion	Local recurrences (vertebrae)/proportion
Breast cancer	46/44.2%	70/49.0%	4/40%
Prostate cancer	16/15.4%	20/14.0%	2/20%
Lung cancer	15/14.4%	21/14,7%	1/10%
Gastrointestinal cancer	10/9.6%	11/7.7%	1/10%
Multiple myeloma	3/2.9%	5/3.5%	0
Renal cancer	3/2.9%	4/2.8%	1/10%
Melanoma	3/2.9%	4/2.8%	0
Sarcoma	3/2.9%	3/2.1%	1/10%
Other gynecological cancers	3/2.9%	3/2.1%	0
Other urogenital cancers	1/1.0%	1/1.4%	0
Others (haemangioma)	1/1.0%	1/0.7%	0

## Discussion

We here report the results for the largest cohort of patients treated with Kypho-IORT to date. The findings for LC for 6 months are in line with earlier results from a smaller cohort with shorter follow-up treated within the phase I/II trial at our institution [9]. The overall excellent control rates after Kypho-IORT compare favorably with data from external beam radiotherapy (EBRT) or stereotactic body radiotherapy (SBRT) [11, 12]. With increasing life expectancy due to improved systemic therapy (including targeted therapies and immunomodulators) and promising therapy options even for patients with metastatic cancer [13], both long-term efficacy and toxicities of local therapies for spinal metastases are gaining further importance.

EBRT, the most widespread standard, offers moderate pain reduction but with the drawback of a delayed onset within several weeks after irradiation, while pain relief in Kypho-IORT is immediate [9, 11]. SBRT to spinal lesions frequently results in flare-up of pain (20–70%) before pain relief but shows higher local control rates than those achieved with EBRT [12, 14–16]. However, a major complication after SBRT is vertebral compression fracture (VCF), which may occur in up to 40% of patients, specifically in case of pre-existing sintering [17–19]. Albeit occurrence or progression of VCF after SBRT resulted in a low mean increase of pain, SBRT may further pose a risk of VCF in adjacent vertebrae [17, 20]. The inherent stabilization and restoration of the vertebral body by cement augmentation virtually eradicates the risk of VCF or further progression of VCF, especially for patients with pre-existing VCF which would have a high probability of progredient VCF after SBRT. Adjacent level fractures after kyphoplasty in patients with (oligo-)metastatic cancer seem to be less likely to occur – in contrast to osteoporotic patients – due to the rather normal bone structure in the vicinity of the treated vertebra which is not affected by the procedure [21]. Since radiation from electronic brachytherapy as part of Kypho-IORT is not only confined to the vertebral body but also of low energy (50 kV X-rays), it can be performed in any operating room suitable for c-arm fluoroscopy [22]. Combining kyphoplasty with radiofrequency ablation instead in regard of radioprotection is therefore not necessary and yielded inferior results in local control [5]. Previous findings showed that kyphoplasty with irradiation can be accomplished in an average time of 65 min and, due to its percutaneous approach, is a surgical intervention, but is considered minimally invasive [9, 23]. Systemic therapy can thus be concomitantly administered without aggravating side-effects (e. g. prolonged wound healing time or increased radiation toxicity).

The limitations of our study, specifically when measuring LC, are the retrospective nature and the heterogeneous histologies and stages (e. g. oligometastatic vs. advanced systemic disease) with patients receiving various other therapies after Kypho-IORT [24].

One could argue that the high LC is—at least partly—aided by systemic therapies since more than half (63%) of the lesions treated in our cohort arose from breast or prostate cancers, where anti-hormonal therapy, anti-resorptive agents, targeted therapies or chemotherapy can effectively contribute to local control at the site of Kypho-IORT [25]. However, also more than half of the recurrent tumors still stemmed from breast (4 patients) or prostate cancer (2 patients) while only 4 patients with recurrent tumors suffered from cancers that are classically less susceptible to systemic therapies (lung, renal, or colorectal cancer, and soft tissue sarcoma) thus suggesting a high innate efficacy of Kypho-IORT independent of histology.

In conclusion, Kypho-IORT resembles a “one-stop-shop” procedure that offers fast pain relief, instant stabilization, and exceptional long-term local tumor control for patients with vertebral metastases.

## Data Availability

The datasets used and analyzed during the current study are available from the corresponding author on reasonable request.
